# Causal Effects of Alcohol-Related Facebook Posts on Drinking Behavior: Longitudinal Experimental Study

**DOI:** 10.2196/28237

**Published:** 2021-11-11

**Authors:** Hanneke Hendriks, Wouter de Nooy, Winifred A Gebhardt, Bas van den Putte

**Affiliations:** 1 Behavioural Science Institute Radboud University Nijmegen Netherlands; 2 Amsterdam School of Communication Research University of Amsterdam Amsterdam Netherlands; 3 Department of Health, Medical and Neuropsychology Leiden University Leiden Netherlands

**Keywords:** social media, social networking site (SNS), alcohol-related posts, alcoholposts, alcohol consumption

## Abstract

**Background:**

Adolescents and young adults frequently post alcohol-related content (ie, alcoholposts) on social media. This is problematic because both social norms theory and social learning theory suggest that viewing alcoholposts of peers could increase drinking behavior. It is therefore paramount to understand the effects of exposure to alcoholposts on viewers.

**Objective:**

This study aimed to investigate the causal effects of exposure to alcoholposts on alcohol consumption by using a rigorous design.

**Methods:**

We conducted a 6-week longitudinal study during which alcoholposts were measured by a newly developed app that copied Facebook posts shared by participants (n=281) to a new social media environment. In addition, daily questionnaires assessed alcohol use. Effects of natural alcoholposts (ie, posted by the participants) were assessed in phase 1, and effects of experimental posts (ie, posted by fake participants) were explored in phase 2.

**Results:**

Results showed that natural alcoholposts increased the occurrence and quantity of drinking the following day. That is, exposure to a single additional alcoholpost increased the log odds of drinking the next day by 0.27 (b=.27, credible interval [CI] .18 to .35). Furthermore, the number of natural alcoholposts had a positive (predictive) effect on the number of glasses drunk the next day (b=.21, CI .14 to .29). In phase 2 when experimental posts were also present, these effects decreased. Experimental posts themselves had hardly any effects.

**Conclusions:**

This study illustrates clear and direct effects of exposure to alcoholposts on next-day alcohol consumption and suggests that alcoholposts represent an important societal problem that interventions need to address.

## Introduction

### Interpersonal Communication About Alcohol Use

Adolescent alcohol abuse is related to severe accidents, brain damage, and future alcohol addiction [1,2]. Studies in offline, face-to-face contexts have shown that interpersonal communication about alcohol influences alcohol consumption [3,4]. However, as a result of significant changes in the interpersonal communication and media landscape, communication today often takes place online [5]. In particular, adolescents and young adults frequently visit social networking sites (SNSs) such as Facebook and Instagram and often post alcohol-related content on these sites [6,7]. Examples of alcohol-related SNS content (henceforth: alcoholposts) are party pictures posted on Facebook in which groups of people are holding alcoholic drinks or Instagram photos in which a close-up of a cocktail is shown [8]. Given the prevalence of alcoholposts, it is paramount to understand the effects of exposure to alcoholposts on their viewers.

Some recent studies suggest that alcoholposts are related to increased alcohol consumption. Unfortunately, however, most of these studies do not allow for strong conclusions about causality because they use cross-sectional designs [6,9-11], making it impossible to conclude whether posting or seeing more alcoholposts leads people to consume more alcohol or vice versa. Furthermore, previous studies frequently rely on retrospective self-reported social media use [8,12-14], which can be problematic because people may have difficulty remembering what they have encountered on social media and at what exact time, especially if the inquired recollection overarches a longer period (eg, in the past year). Last, not many studies have experimentally investigated effects of alcoholposts in a realistic social media environment. Thus, to ascertain the effects of alcoholposts on alcohol use, there is an urgent need for longitudinal studies that combine daily measurements of alcohol consumption with daily objective measurements of alcohol-related social media content and that experimentally study the effects of alcoholposts on drinking behavior.

### Alcohol-Related Content on Social Media

The rise of social media introduced new ways for young people to communicate with each other. A multitude of studies examining platforms such as Facebook, Instagram, and YouTube show that young people post many alcoholposts. Although estimated percentages of young people posting alcoholposts vary between 36% to 96%, studies agree that alcohol is frequently visible on SNS [6,7,15]. Most adolescents and young adults indicate that they post alcoholposts because they want to entertain others or celebrate and share nice moments with friends [16]. Furthermore, it has been suggested that posting alcoholposts is something that people are generally not consciously aware of, as it often happens without deliberate intent [17].

Content analyses have drawn several important conclusions about the nature of alcoholposts: (1) they mostly portray alcohol use as a normal part of life (eg, pictures of dinners or parties), (2) they almost always involve a positive context (eg, laughing faces), and (3) they often contain a social component (eg, showing groups of people) [8,18,19]. All 3 aspects are problematic. The first 2 because they imply that alcoholposts express to viewers that alcohol use is positive and normal, while at the same time the negative consequences of alcohol use (eg, drunk people embarrassing themselves) are ignored. The third aspect is also alarming, because a vast body of research consistently confirms that social norms are very powerful in influencing behavior [20-23]. Thus, viewing alcoholposts in which many people are drinking alcohol is likely to have a strong influence on behavior. Given these characteristics and the presence of alcoholposts on social media, it is vital to better understand how viewing such posts influences drinking behavior.

### Why Viewing Alcoholposts May Make People Drink

The tenets of both social norms theory [24] and social learning theory [25] would predict that seeing alcoholposts strongly increases drinking behavior among viewers. Social learning theory and later social cognitive theory [26] posit that behaviors can be learned from observing and imitating others. Moreover, it is suggested that especially seeing behavior rewarded or punished can lead to learning effects and behavior changes. As argued, alcoholposts are often positive and social (eg, showing laughing people being present at fun events). These aspects of alcoholposts may make alcohol use seem rewarding, leading viewers to increase their drinking behaviors.

Social norms theory similarly suggests that behavior is influenced by people’s perceptions of how others think and act. These perceived norms are more important for behavior than objective norms (ie, what others actually do), while they are likely to be based on misperceptions of how others behave [27]. In the context of alcohol use, it has been shown that people often mistakenly think that others drink more alcohol than they do themselves (ie, pluralistic ignorance [28]). This false belief may be reinforced by exposure to alcoholposts in which seemingly everyone is engaging in drinking behaviors. Such a belief may particularly increase drinking behaviors in viewers who want to fit in and behave in line with existing norms (see also Beullens and Vandenbosch [29]).

### Studies on the Relationship Between Alcoholposts and Alcohol Consumption

Thus, in line with both social norms theory and social learning theory, viewing alcoholposts of peers on social media is expected to increase drinking behavior. Several recent studies have addressed this relationship; however, some important aspects of these studies limit the implications of this previous research.

#### Cross-sectional Studies

Several cross-sectional studies suggest that exposure to alcoholposts is related to drinking behaviors [9,10]. For example, Ranney et al [30] linked state-wide alcohol tweets with emergency care visits. Geusens and Beullens [6] observed that self-reported alcoholposts on social media were related to increased self-reports of alcohol abuse, and Thompson and Romo [11] found that self-reported alcoholposts were associated with alcohol-induced problems. However, in these studies questionnaires measured both seeing alcoholposts in a previous period and alcohol use in the previous period. Therefore, it is not clear whether alcoholposts predict drinking or are a consequence of (ie, simply reflect) it.

#### Longitudinal Studies

A handful of recent studies have used a longitudinal design to address the relationship between exposure to alcoholposts and alcohol use. For example, Tucker and colleagues [15] showed that greater self-reported exposure to alcoholposts in 7th grade predicted drinking in 8th grade, controlled for drinking at 7th grade. Erevik and colleagues [31] showed that especially self-reported disclosure (instead of exposure) of alcoholposts predicted alcohol use 1 year later. Furthermore, in a study by Boyle et al [12], self-reported exposure to alcoholposts during the first 6 weeks of college predicted alcohol use 6 months later, controlled for previous alcohol use. However, these longitudinal studies all measured exposure to alcoholposts by using self-report (eg, questions such as “How often during the past 3 months did you see pictures on an SNS showing or talking about someone who is drunk?” [9]). This is problematic, since evidence suggests that it is very hard to correctly recall media exposure (ie, recall bias), especially if it covers a longer time period in the past [13,14]. The problem of self-reported media exposure is not unique to this context and poses a challenge for researchers in many other related fields [32].

Furthermore, these previous studies used a long time span between exposure to alcoholposts and alcohol use (eg, 6 or 12 months), whereas a short time span (eg, 1 day) would provide useful insights into the direct effects of viewing an alcoholpost on drinking shortly afterward. That is, although longitudinal designs with longer time spans may provide information on long-term effects of alcoholposts, it is unclear what occurs exactly in between waves, making the direct and short-term effects of alcoholposts unknown. Thus, although a few valuable longitudinal studies exist, limitations in their designs curtail our understanding of the direct causal effects of alcoholposts on alcohol use. In this study, we applied a longitudinal design in which we measured exposure to alcoholposts objectively and included daily measurements of exposure to alcoholposts as well as alcohol use.

#### Experimental Studies

Not many experimental studies exist that address the effect of exposure to alcoholposts on alcohol use. One experimental study by Alhabash et al [33] manipulated various aspects of alcohol marketing posts and studied subsequent effects (see also Alhabash et al [34]). In line with this, another experimental study by Noel [35] on alcohol marketing manipulated types of ads as well as comments and studied effects on purchase intentions. Although very valuable, these studies focused on alcohol marketing and not on user-generated content. Furthermore, these studies did not use a realistic immersive social media environment reflecting real-life conditions. We argue it is essential to use an experimental design to study the effects of alcoholposts in order to be able to make inferences about causality. That is, if participants are randomly assigned to different alcoholpost conditions, then any differences between groups with regard to drinking behavior can be attributed to the exposure to the specific posts within the experimental conditions. Therefore, to solidify our claims about the causal effects of alcoholposts, we also used an experimental design in which the effects of experimentally manipulated exposure to alcoholposts were assessed. We did this in a realistic social media environment closely resembling real life.

### Our Study

In sum, although previous studies have provided valuable insights into alcohol-related social media use, to provide a clear answer about the causal effects of exposure to alcohol content on social media, we used a longitudinal study that combined objectively measured daily exposure to alcoholposts with daily measurements of alcohol consumption (ie, whether [occurrence] and how much [quantity] people drink). Because previous research has shown that existing alcoholposts are mainly positive and social and social norms theory, social learning theory, and empirical evidence suggest that seeing alcoholposts leads to increased drinking behavior, we expected the following:

- H1: Exposure to natural alcoholposts increases the occurrence and quantity of alcohol consumption.

Furthermore, in a second phase of our study we experimentally investigated the effects of alcoholposts. More specifically, existing alcoholposts were observed in the first phase (ie, natural posts), and additional alcoholposts were experimentally manipulated during the second phase (ie, experimental posts). The experimental posts differed in terms of valence (negative versus positive about alcohol) and the degree to whether they were social (ie, showed people), and we investigated how these experimental posts influenced daily measures of alcohol consumption. Because previous research [21,36] has shown that the activation of positive alcohol associations (eg, as a consequence of positive alcoholposts) and the observation of other people drinking alcohol (eg, as a consequence of social alcoholposts) can increase alcohol consumption, we expected the following:

- H2a: Exposure to positive experimental alcoholposts increases the occurrence and quantity of alcohol consumption, whereas negative experimental alcoholposts decrease the occurrence and quantity of alcohol consumption.
- H2b: Exposure to social experimental alcoholposts has a stronger influence on the occurrence and quantity of alcohol consumption than exposure to nonsocial alcoholposts.

In this study, we focused on college students, based on a previous study highlighting that college students (aged 18 to 30 years) posted far more alcoholposts than high school students (aged 12 to 18 years) [17]. Furthermore, we chose to focus on Facebook because this was the most popular social media channel among our target group (college students in the Netherlands) at the time the study was conducted. That is, 89% of Dutch people aged 20 to 39 years used Facebook, in comparison with 46% who used Instagram and 32% who used Snapchat [37]. Among those aged 15 to 19 years, Facebook use was also very high (72%), although Instagram (73%) and Snapchat (72%) were starting to get more users. Furthermore, several studies conducted in this context showed that alcoholposts were common on Facebook. For example, Van Hoof et al [38] showed that 99% of college student Facebook profiles contained alcohol references. Therefore, we used Facebook to study effects of alcohol content.

## Methods

To obtain daily exposure measurements of alcoholposts, we developed a social media app that copied participants’ Facebook posts to a new and realistic social media environment (more information below). Effects were assessed on daily measurements of alcohol consumption.

### Participants

Participants were all students and participated in groups (ie, they were asked to sign up as a group: friends, classmates, colleagues). Most groups were friends or classmates. The reason for recruiting in groups is that we wanted participants to know some but not all of the other participants. That is, we wanted them to see posts of people who were familiar to them (as is normally the case on social media), but we also wanted them to see posts of people unfamiliar to them. That way, we could add experimental posts of fake unfamiliar participants to the study without participants’ awareness. In all analyses we controlled for group (ie, we did multilevel analyses).

The baseline survey was answered by 306 participants. During the course of 6 weeks, 25 participants never answered the daily survey and were therefore omitted from analyses. Therefore, 281 participants, who were part of 49 groups, were included in the analyses (208 women, 73 men, mean age [SD 1.90] 20.53 years, range 17 to 30 years). All participants were Dutch. In the Netherlands, the minimum legal age to purchase alcoholic beverages is 18 years.

There were 49 groups in total. Group sizes ranged from 2 to 18 participants. Most groups consisted of 4 to 5 people. That is, 7 (14%) groups had 2 to 3 participants, 25 groups (51%) had 4 to 5 participants, 8 groups (16%) had 6 to 7 participants, 4 groups (8%) had 8 to 9 participants, and 5 groups (10%) had 10 to 18 participants. At the beginning of the study, there were 722 existing Facebook friendships within these groups (out of 922 possible friendships within groups). Across groups, there were 653 friendships (out of 38,418 possible friendships across groups). On average, a participant had a Facebook friendship with 4.6 participants from another group.

### Design

The study used a longitudinal design with 43 measurements (1 baseline survey and 42 daily measurements). Participants first completed a baseline survey and starting 1 week later, they were followed for 6 weeks (ie, 42 days in total) during which alcohol-related social media use and alcohol consumption were measured on a daily basis.

The study had 2 phases: during the first 3 weeks, posts were merely observed (ie, natural posts), and during the next 3 weeks, fake participants (ie, profiles made by the experimenter) posted additional alcoholposts (ie, experimental posts). In order to test the influence of different types of experimental posts, we manipulated 2 aspects of the experimental posts. That is, based on studies that show that alcoholposts are usually positive and social [8,19], we manipulated whether the post had a negative or positive context and also whether the post was social or not social (ie, no people visible). Groups were randomly assigned to 1 of the 4 conditions in this 2 (negative versus positive) × 2 (not social versus social) between-subjects design. During phase 2, participants only saw experimental posts within the same condition (eg, only positive social experimental posts).

### Procedure

Participants were recruited at university campuses and student buildings and were asked to participate in groups (minimum size of 4 people). At the beginning of the baseline survey, participants were informed about what their participation in the study entailed (ie, completing daily questionnaires, engaging with the SNS app, and giving access to their Facebook posts) after which they provided their informed consent.

To prevent participants from correctly guessing the purpose of the study, they were told that they would be involved in 2 separate study parts (ie, 1 focusing on the questionnaire, and 1 focusing on Facebook using the SNS app). Next, they answered questions concerning demographics and expected covariates (ie, gender, study year, habitual frequency of alcohol use, and habitual quantity of alcohol use). At the end of the baseline survey, participants received instructions on how to download, install, and use the SNS app.

A week thereafter the study started. Participants took part in the study for 6 weeks. Every day, they had 2 tasks: answer a short questionnaire (measuring alcohol use on the previous day) and visit and engage with the SNS app. Filler questions (about exercising and snacking behaviors) were added to the questionnaire to obscure the real purpose of the study. To help remind participants, we sent push messages with a link to the questionnaire via the SNS app every day at 9 AM. Furthermore, participants could click on the SNS app, view posts, and engage (ie, like and/or comment) with posts (more information on the SNS app is described later). Participants were told that it was very important to answer the questionnaire and visit the app every day. We monitored whether participants opened and engaged with the SNS tool (time was not monitored). When participants did not log in, they would receive a reminder to engage with the tool. In the beginning, this was done for everyone on a daily basis. After a few weeks, ensuring that participants were engaging actively, this was done by randomly checking activity levels of participants.

As stated, the study had 2 phases: during the first 3 weeks (days 1 to 21), posts were merely observed. That is, only real posts (ie, natural posts) of the participants were visible in the app. During the next 3 weeks (days 22 to 43), fake participants (ie, profiles made by the experimenter) posted additional alcoholposts (ie, experimental posts; based on the 4 between-subject conditions).

After 43 days, participants answered the final survey after which they were debriefed and rewarded (€30 [US $35] per participant). All participants were extensively debriefed, especially on the fact that there were (positive) fake experimental posts in the second phase and that they should be aware of the negative consequences of alcohol use. At any time, participants were allowed to withdraw their participation, also after reading the debrief (and what the study was really about). The study underwent extensive ethical screening and received ethical approval by the University of Amsterdam’s ethical board (2018-PC-8731).

### Materials

#### SNS App

**Figure 1:**
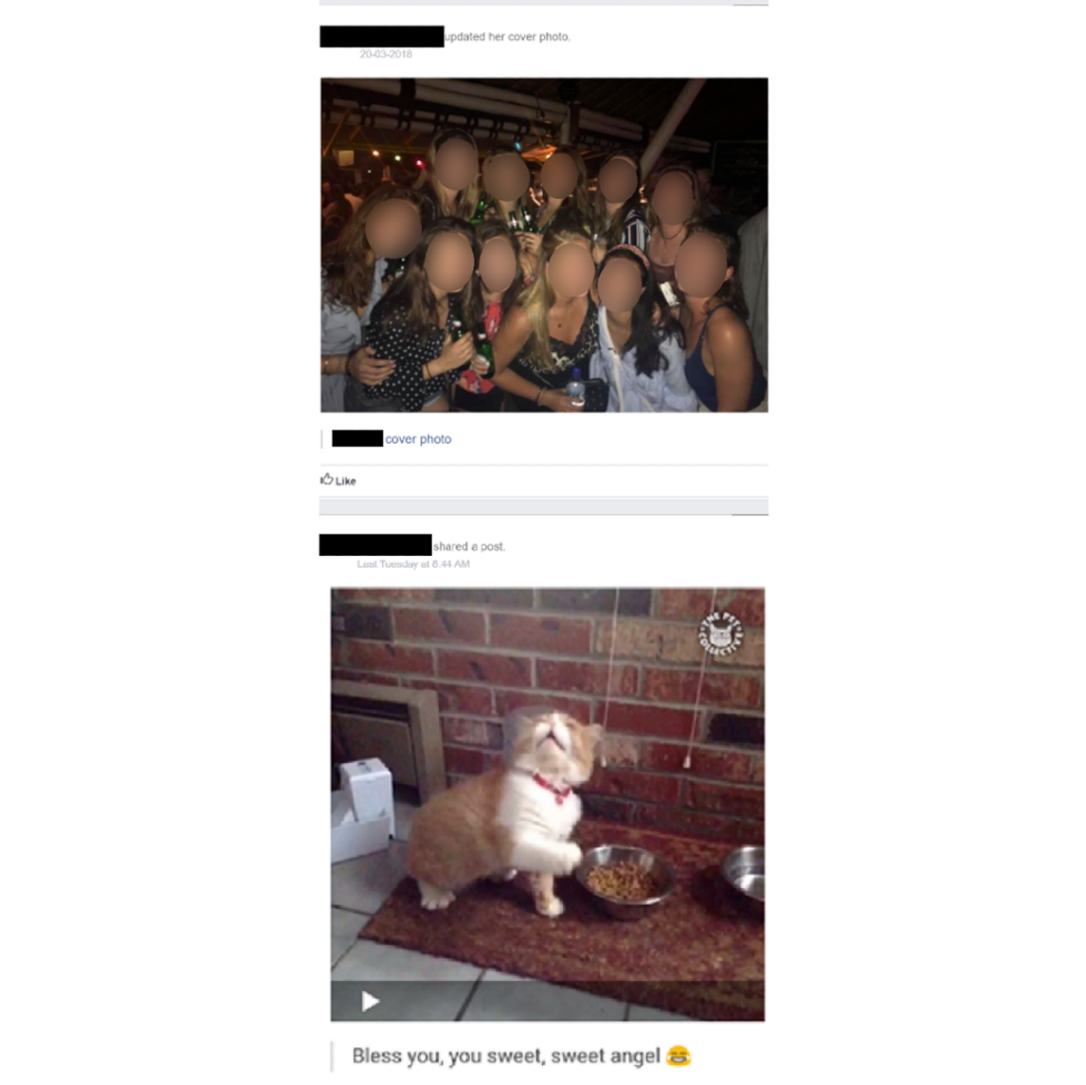
Visual representation of the timeline of the social networking site tool.

To measure social media use, an app was developed for this study by the software company Akyla. After participants downloaded and accepted the terms of the app, it was able to copy all posts that participants posted on Facebook from that moment on. These posts were subsequently posted in the app, which strongly resembled a Facebook environment. See Figure 1 for a visual representation of the timeline. In this app, participants were able to see their and other participants’ posts and engage (ie, like and/or comment) with them in a similar way as can be done on Facebook. Using this app had several advantages over using Facebook directly: (1) we were absolutely sure what posts and in what order participants were exposed to in the app (with current Facebook algorithms, this is not transparent and individual-specific), (2) we only showed participants posts of other participants (and not of friends of friends) and we only collected participants’ posts and not posts of their friends who did not participate in this study, thereby decreasing privacy concerns, and (3) we were able to add experimental posts to this SNS context in a realistic way.

#### Natural Posts

All posts posted in the SNS app were automatically stored for the experimenter in an Excel (Microsoft Corp) file. This Excel file was coded in order to determine whether posts were alcoholposts based on a coding book by Hendriks et al [8]. If a post clearly showed alcohol in the picture or clearly referred to alcohol in the header, it was coded as an alcoholpost. If this was the case, it was also coded whether the context of alcohol was positive (eg, showing laughing people or positive consequences of alcohol use [having fun]), negative (eg, showing frowning people or negative consequences of alcohol use [a hangover]), or neutral (ie, when it was not clearly positive or negative), and whether the alcoholpost was social or not (showing people versus no people visible).

#### Experimental Posts

**Figure 2:**
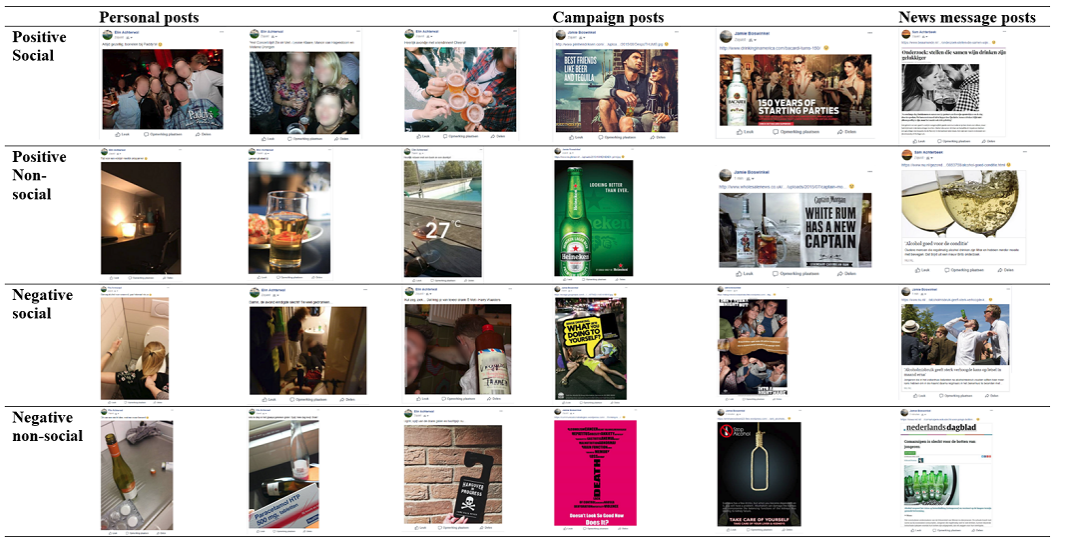
All experimental posts used during phase 2.

There were 24 experimental posts in total. Participants saw 6 experimental alcoholposts within their allocated condition distributed over a period of 3 weeks. All posts fit the condition; however, we used different types of posts to make the manipulation less obvious and provide more variation. That is, we used 3 personal posts, 2 campaign posts, and 1 news post. The personal posts were similar to the majority of alcoholposts previously reported in literature [8] and showed personal photos of experiences (eg, a night out). The campaign posts reflected posts by professional organizations, either being existing alcohol commercials (in case of the positive conditions) or existing antialcohol campaigns (in case of the negative conditions). The news posts were existing news messages about, depending on the condition, the negative or positive effects of alcohol use. The 24 experimental posts were based on an extensive pilot study (n=41, 29 women, 12 men, mean age [SD 3.23] 22.90 years, range 18 to 36 years), in which 54 posts were evaluated. Pilot participants evaluated the posts on a Likert scale from 1=very negative to 7=very positive by answering questions related to valence (“I think this post is very negative/very positive about alcohol” and “I find it likely that the person posting this is very negative/very positive about alcohol”) and social aspects (“How many people did you see/tagged in the post?” and “Do you consider this to be a ‘social’ post?” and “Did the post include an individual or social activity?”). Of these posts, the most clearly negative/positive and social/nonsocial posts were chosen. See Figure 2 for all experimental posts used and Multimedia Appendix 1 for translations.

### Measures

#### Daily Alcohol Consumption

Alcohol consumption was measured in the short daily questionnaire by addressing occurrence (whether people drink) and quantity (how much they drink). Occurrence was measured by the question “Did you drink alcohol yesterday?” (no/yes), and quantity was measured by “How many alcoholic drinks did you consume?” (mean 1.57 [SD 3.27] glasses, range 0 to 50 glasses).

#### Filler Questions

In the daily questionnaire, filler questions were asked about exercising and snacking behavior to make the focus on alcohol less obvious.

#### Covariates

The covariates habitual frequency of alcohol use (ie, “How often do you normally consume alcohol?” 8-point scale ranging from “I never drink” to “once a day or more often”), habitual quantity of alcohol use (ie, “On a drinking day, how many alcoholic drinks do you usually have?” 10-point scale ranging from “1” to “10 or more”), gender, and study year were measured in the baseline survey.

### Analyses

#### Multilevel Modeling

We tested multilevel models with 3 levels (ie, daily alcohol use reports [level 1] nested within participants [level 2] nested within groups [level 3]). To examine the effects of alcoholposts on alcohol occurrence, a logistic regression was conducted, and to investigate effects on alcohol quantity, a negative binomial model was tested. We used the Hamiltonian Monte Carlo estimation as implemented in the R package rstanarm [39]. We used a Bayesian approach (Multimedia Appendix 2) to data analysis [40], enabling us to draw conclusions about the probability that a parameter is in a particular range (ie, credible interval). The reader may use this interval for testing null hypothesis significance by checking if the interval contains zero, but we prefer to interpret the intervals because null hypothesis significance testing is highly contested [41,42].

#### Random Effects

Alcohol drinking is usually a habit linked to particular days in the week [43]. In our sample, we observed clear differences between the weekdays in terms of the number of participants who drank alcohol. In addition, we may expect that students have their own individual habitual drinking days. A separately estimated multilevel model with days nested within participants suggested that participants varied in terms of the weekdays on which they drink alcohol. The fact that participants had different preferred drinking days may compromise the causal interpretation of effects of alcoholposts on drinking probability and drinking quantity. That is, alcoholposts are likely to show alcohol use that occurred on the posting day or the preceding day. Imagine that participants have different typical drinking days: for example, participant 1 tends to drink on Thursdays and participant 2 drinks on Fridays. Participant 1’s Thursday drinking may result in an alcoholpost that precedes the Friday drinking of participant 2; however, the alcoholpost does not cause a higher probability or quantity of drinking for this habitual Friday drinker. For this reason, it is important to control for each participant’s individual drinking probability and quantity on each day of the week. In multilevel modeling terms, we included varying (ie, random) effects of the day of the week at the participant level. Effects of exposure to alcoholposts represent changes in the occurrence or quantity of alcohol drinking in comparison to what we normally expect for a participant on this day of the week. In other words, exposure effects show if participants drink more or more often than they normally do on this day of the week.

#### Random Intercepts

Furthermore, we found that there was variation in average alcohol use and quantity across groups and participants. Some groups drink more often or more glasses of alcohol than other groups. The same applies to participants, where individual drinking differences were visible. Therefore, for both the participant and group, we used random intercepts.

#### Covariates

The models controlled for the day of the week and for 4 characteristics of the participants: gender (female: no=0), study year, habitual frequency of alcohol use, and habitual quantity of alcohol use (reported during the baseline survey).

## Results

### Descriptives

Across the 6 weeks of the study, the response rate to complete the daily questionnaire among the remaining 281 participants was 80.8%. Although participants were stimulated to use the SNS tool daily, some days they did not engage with the app. On average, participants used the app on 24 out of 43 days.

There were 547 posts in total. In phase 1, 271 participants shared a post (approximately 1 post per participant), and in phase 2, 194 participants posted something (0.7 post per participant). A total of 39 posts (15 posts in phase 1 and 24 posts in phase 2) were natural alcoholposts, which were posted on 27 separate days. These alcoholposts were posted by 14 participants in phase 1 and 22 participants in phase 2. Focusing on exposure to alcoholposts, the mean number of alcoholposts seen on the previous day was 8.5 (phase 1) and 13.8 (phase 2). There were 7 participants in phase 1 (2.4%) and 15 participants (5.3%) in phase 2 who never viewed an alcoholpost on the previous day. Based on these numbers, we can conclude that almost all participants were exposed to alcoholposts and that this happened frequently.

Coding of these posts revealed that 26 showed a positive context, 11 were neutral, and 2 showed a negative context. Furthermore, 34 of these posts were social (ie, showing people) and 5 were nonsocial (no people visible).

We had exposure measurements for all days except the first day of the observation period. This left us with 8794 alcohol reports (level 1) submitted on 41 days by 281 participants (level 2) who were assigned to 49 groups (level 3). All cases had a valid score on whether the participant drank alcohol; 5 cases had a missing or invalid score for the number of glasses of alcohol consumed and were dropped from the analysis of alcohol quantity consumption.

### Hypotheses Testing

#### Effects of Natural Alcoholposts

We assumed that natural alcoholposts may affect the occurrence of drinking alcohol on the next day and the quantity of alcohol drunk on the next day (H1). The number of alcoholposts created on the day preceding the day with reported alcohol use is our indicator of exposure to alcoholposts. In our model, we tested the effects of natural alcoholposts for the entire period (phase 1 and phase 2); however, we added phase as a moderator so that we could determine the effects of natural posts for each phase separately and also formally test the interaction effect.

#### Phase 1

We started our analyses by looking at phase 1 (ie, the phase without experimental posts). Results showed that in phase 1, the number of natural alcoholposts had a positive (predictive) effect on the probability of drinking the following day. Exposure to a single additional alcoholpost increased the log odds of drinking the next day by 0.27 (b=.27, credible interval [CI] .18 to .35). This means that, for example, seeing 1 instead of no alcoholpost the day before increased the chance of drinking alcohol from 40% to 47%. Seeing 4 instead of no alcoholposts on the preceding day increased the chance of a drinking day to 66%. (This example is based on drinking on a Saturday for a female, first-year student with average scores on other predictors).

Furthermore, not only occurrence but also quantity of alcohol use was predicted by alcoholpost exposure. That is, the number of natural posts also had a positive (predictive) effect on the number of glasses drunk (b=.21, CI .14 to .29). Using the same example as before, this means that seeing 1 instead of no alcoholpost the day before increased the number of alcohol beverages consumed from 1.64 glasses to 2.03 glasses. Seeing 4 instead of no alcoholposts on the preceding day further increased the number of alcohol beverages consumed to 3.86 glasses. Therefore, H1 is supported for phase 1.

#### Phase 2

**Figure 3:**
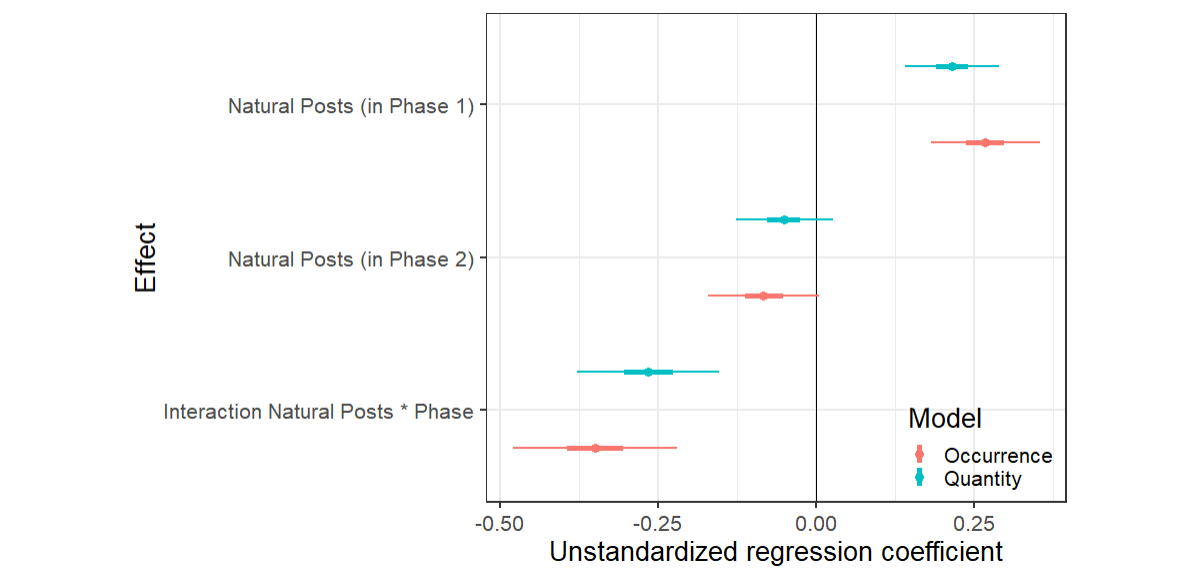
Predicted effects of natural alcoholposts per experimental phase and their 95% and 50% credible intervals for the model predicting occurrence and the quantity of alcohol consumed.

**Figure 4:**
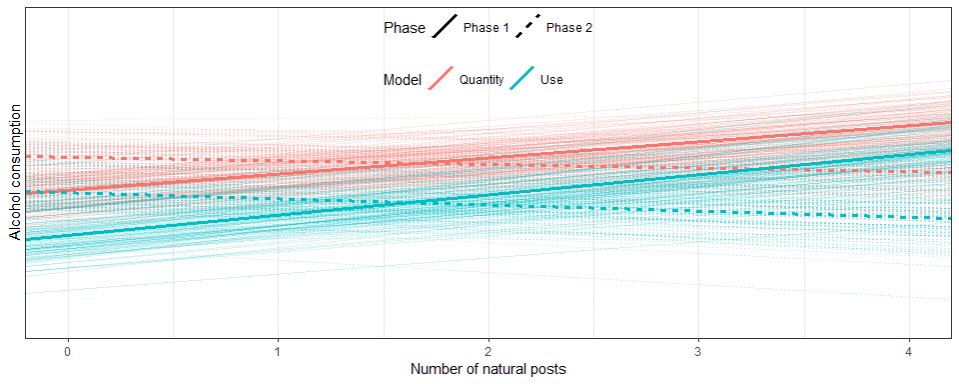
Regression lines for the effects of natural posts on alcohol consumption (occurrence and quantity) in phase 1 and phase 2. The different slopes of the bold and dashed lines illustrate the interaction effect.

In phase 2, experimental posts were added to the app. As these additional posts may have affected the influence of natural posts, we estimated whether the influence of natural posts depended on the phase (ie, an interaction effect). Indeed, the analyses showed that there is an interaction effect when predicting the probability of drinking (b=–.35, CI –.48 to –.22) and when predicting the number of glasses drunk (b=–.27, CI –.38 to –.15), suggesting that the effects of natural posts depended on the phase. Indeed, although we found a positive effect of natural alcoholposts on alcohol use in phase 1, we saw no convincing positive effects of natural alcoholposts in phase 2 on occurrence (b=–.08, CI –.17 to 0.1) or quantity (b=–.05, CI –.13 to .03). See Figure 3 for all credible intervals and Figure 4 for an illustration of this interaction effect.

#### Effects of Experimental Posts

H2a predicted that positive experimental alcoholposts lead to increased alcohol occurrence and quantity, whereas negative experimental alcoholposts lead to lower alcohol occurrence and quantity. Results strongly suggest that positive experimental posts have small positive unstandardized regression weights when predicting occurrence (b=.06, CI –.02 to .14, posterior probability of a positive effect is .933), but we are less sure of a positive effect on quantity (b=.02, CI –.05 to .09, posterior probability of a positive effect is .710). Although this suggests that positive experimental posts are more likely to have a positive than negative effect on the occurrence of alcohol use, we are not sufficiently sure about an effect because these credible intervals contain zero.

Looking at negative posts, the effects become even more uncertain and their directions are contradictory. That is, negative experimental posts have small positive unstandardized regression weights when predicting occurrence (b=.02, CI –.06 to .10) and small negative rather than positive unstandardized regression weights when predicting quantity (b=–.02, CI –.10 to .05). These results do not allow us to draw a conclusion about the effects of negative experimental posts. We therefore find little support for H2a.

H2b predicted that social experimental alcoholposts had stronger effects on occurrence and quantity than nonsocial posts. Results showed that social experimental posts have small positive unstandardized regression weights when predicting occurrence (b=.05, CI –.03 to .14); however, this was not the case when predicting quantity (b=.00, CI –.08 to .08). Furthermore, nonsocial alcoholposts have small regression weights when predicting alcohol occurrence (b=.02, CI –.06 to .11) and quantity (b=.00, CI –.07 to .07). Although this suggests that social experimental posts are more likely to have a positive than negative effect on alcohol use and these effects seem larger than those from nonsocial posts, we are not sufficiently sure about the effects of social posts because these credible intervals contain zero. Thus, we do not see strong support for H2b.

#### Type of Posts

As shown in Methods, we used 3 types of experimental alcoholposts (ie, personal posts, campaign messages, and news messages). It is possible that some of these posts are more influential than others, potentially explaining the varying effects of experimental posts described above. Therefore, we exploratively tested whether these types of experimental posts have different effects.

**Figure 5:**
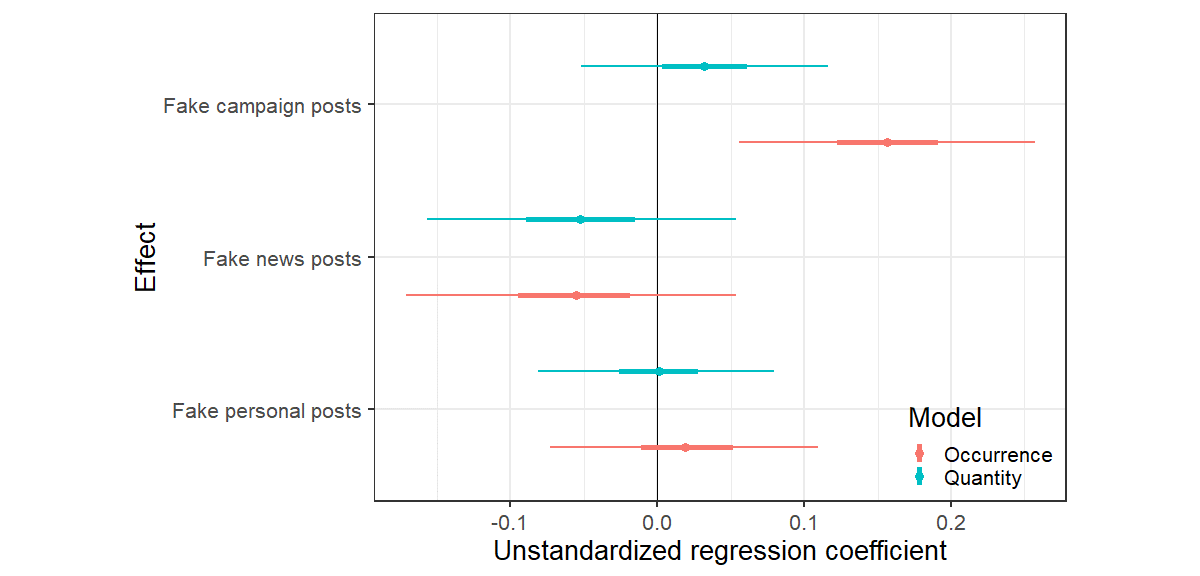
Predicted effects of experimental campaign, news, and personal alcoholposts and their credible intervals for both models predicting occurrence and quantity of alcohol consumed.

As can be seen in Figure 5, personal or news posts did not hold clear negative or positive effects on alcohol occurrence (b personal=.02 , CI personal –.07 to .11; b news=–.06, CI news –.17 to .05) or quantity (b personal=.00, CI personal –.08 to .08; b news=–.05, CI news –.16 to .05). However, experimental campaign posts (ie, both proalcohol commercials and antialcohol campaigns) had a positive effect on alcohol occurrence (b=.16, CI .06 to .26), although not on quantity (b=.03, CI –.05 to .12). An additional campaign post increases the odds of a drinking day on the next day by 16%. Using the same example as before but now focusing on the second phase, this means that seeing 1 experimental campaign post instead of no experimental alcoholposts on the preceding day increased the chance of drinking alcohol from 53.9% to 57.8%, and seeing 3 campaign posts increased the chance of a drinking day to 65.2%. See Figure 5 for the credible intervals. Please see Multimedia Appendix 3 for additional tables outlining our results, including the influence of control variables.

## Discussion

### Principal Findings

Given that alcoholposts are often present on social media and have potentially undesirable effects on alcohol use, the purpose of this study was to investigate the causal effects of exposure to alcoholposts on alcohol consumption. We conducted a longitudinal study that combined daily measurements of alcohol consumption with objectively measured daily measurements of alcohol-related social media content and that in a second phase also experimentally studied the effects of alcoholposts on alcohol use. Our analyses provide 3 main findings: (1) alcoholposts increase the occurrence and quantity of alcohol consumption on the next day, (2) these effects of alcoholposts disappeared in the second phase of the study when the experimental alcoholposts were posted, and (3) the experimental alcoholposts had hardly any effect on drinking behavior.

The first finding was that exposure to an alcoholpost increased the chance of drinking alcohol as well as the number of alcoholic beverages consumed that following day. Thereby, this study suggests a direct causal effect of exposure to alcoholposts on proximal (next day) alcohol consumption. Although several studies have explored this relationship in a cross-sectional [6,9-11] or longitudinal way [eg, 12], limitations in these designs have restricted the conclusions that could be to drawn on the causal direct effects of alcoholposts. By using daily alcohol consumption measures and daily objective measures of alcoholposts, this study shows that if young people encounter alcoholposts in their social media environment, this increases the chance that and how much they drink the next day.

The second finding was that the effect of exposure to alcoholposts depends on the phase of the study. Whereas in phase 1 the effects of natural alcoholposts on alcohol use were very clear, there were hardly any effects of these alcoholposts visible in phase 2. A potential explanation for this may be that the addition of the experimental posts (6 for each condition) influenced the impact of the natural alcoholposts. Previous research confirms that alcoholposts are positive and social [18,19]. By adding negative and nonsocial posts, we have provided a more diverse alcohol-related social media environment that is not necessarily all proalcohol, which might have dampened the undesirable effects of natural alcoholposts. If this is indeed the case, this provides important ideas for interventions, as this suggests that adding antialcohol content to a social media environment decreases the undesirable impact of alcoholposts. Future research is necessary to investigate whether this indeed is the case.

The third main finding was that exposure to the experimental alcoholposts had almost no effect on drinking behavior. That is, negative experimental posts did not decrease alcohol use, positive alcoholposts did not increase alcohol use, and neither did social posts differ in effects from nonsocial posts. This was quite surprising, as the experimental posts were based on existing alcoholposts and subjected to an extensive pilot study. At first glance, a possible explanation why natural posts had more effects than experimental posts is related to familiarity. That is, the natural posts in our study were posted by real individuals (and experimental posts were posted by fake individuals), making it more likely that the posters were known by other participants. However, we would like to highlight that participants were part of 49 groups, and they were familiar with on average only 4.6 participants from other groups (see Methods). Thus, natural alcoholposts very often were placed by strangers as well. Therefore, we do not think that familiarity can fully explain the differences between experimental and natural posts.

An alternative explanation for why we found almost no effects of experimental posts may be because some of the alcoholposts we used (eg, news posts about alcohol) were not very common in a real social media environment. However, we did find an effect of 1 type of experimental alcoholpost: campaigns. Experimental campaign posts increased whether people drink the next day (but not how much they drink). Interestingly, however, campaigns increased the chance of a drinking day regardless of whether this campaign post was positive about alcohol (ie, an alcohol commercial) or negative about alcohol (ie, an antialcohol campaign). A possible explanation may be that seeing alcohol on social media can serve as a prime and may trigger existing alcohol-related associations that are positive (eg, alcohol is fun [36]), even if alcohol is portrayed negatively. Another explanation may be that antialcohol campaigns increased drinking because of psychological reactance [44]. That is, when individuals feel threatened in their freedom (eg, when a campaign suggests that they should drink less), this may cause reactance against the message. These explanations for this undesirable effect need to be tested in future studies because this may suggest that using antialcohol campaigns on social media may not be a wise strategy.

The purpose of this study was to provide insight into the causal effects of alcoholposts on alcohol use. Because our findings confirm effects of natural alcoholposts but show hardly any effects of experimental posts, we can be relatively sure about order effects but still need more research to be fully certain of the causality of effects. That is, by showing that alcoholposts predict next day drinking and by controlling for personal drinking rates, we show that there are direct acute relations between seeing an alcoholpost and drinking (instead of the other way around). However, we cannot exclude the possibility that third variables exist that relate to both alcoholposts and drinking (eg, alcohol-related events). We therefore recommend additional experimental research manipulating alcoholposts to provide even more clarity on the causality of effects.

### Practical Implications

The most important finding of this study is that exposure to alcoholposts increases whether and how much college students drink. Given the abundance of alcohol-related content on social media [6,9], this is a worrisome conclusion. Previous studies have shown high percentages (ie, between 36% and 96%) of young people having alcoholposts on their profile. Our study shows a different perspective on this percentage: that is, we counted 36 respondents (13%) who posted at least 1 alcoholpost, a percentage that is a much lower than those mentioned in previous literature. A potential explanation is that our study is unique in using a short timespan to study the direct effects of alcoholposts. Previous studies have often coded posts in a period of a year or by even coding entire profiles existing of many years. It is therefore not surprising that the latter strategy would yield more alcoholposts than the former. If our study would have focused on a period of 1 year instead of 6 weeks, the percentages found would probably be more in line with previous research.

Although the number of alcoholposts was relatively low in our study, the 39 alcoholposts reflect a large number of people who are exposed to the alcoholposts. On the 43 days of this study, there were 39 instances in which participants saw an alcoholpost. As stated, 1 alcoholpost can already increase the occurrence and quantity of drinking, meaning that exposure to 39 alcoholposts can have a big impact on drinking behavior. We believe this is the crux of the problem of alcoholposts: a single alcoholpost on social media may have enormous reach and simultaneously affect hundreds of people. This problematic impact of alcoholposts becomes even worse if the person posting the alcoholpost is popular and has a large number of friends or followers (ie, is a social influencer [45]), thereby highlighting the need to address this urgent societal issue.

The question then is how to tackle this problem? Although this was not the purpose of the study, we propose 2 potential ways to approach this issue: decrease the posting of alcoholposts and decrease the unhealthy effects of alcoholposts. Many strategies might or might not work in this regard, and future studies need to explore these intervention ideas. To address the first issue, one might need to make young people aware that alcoholposts can pose a real problem and have a negative impact. Also, one could highlight that other people (eg, future employers or parents) might negatively evaluate posters of alcoholposts, or—as people are often not consciously aware that alcohol is visible in their posts—one could implement automatic warnings on social media when people are about to post an alcoholpost that state “You are about to share a post in which alcohol is visible. Are you sure you want to do that?”

To address the second issue, one could make the social media environment more heterogeneous by adding negative alcohol content. However, based on our finding regarding experimental campaign posts, one should be mindful of the type of negative alcohol content chosen for this purpose. Stimulating peers to also post negative alcohol experiences (eg, hangover posts) might be a possibility. It could also be an idea to stimulate negative comments to alcoholposts or have people withdraw their tags from alcoholposts. Doing so might decrease the normative beliefs in young viewers that alcohol is normal and positive [27-29].

An alternative approach would be to illustrate the “fake” nature of alcoholposts (eg, by showing an alcoholpost with the caption “What you think happened?” next to which an alcohol photo is shown in which a person is lying drunk on the ground with the caption “What happened after”). Future research is needed to test whether these ideas have desirable public health outcomes.

### Limitations

Although our study design had several strengths, some limitations should also be noted. First, even though our study used objective social media measurements, alcohol use was measured through self-report. The reason this was done was that measuring alcohol consumption in objective ways (eg, through breathalyzers or observations [46]) for 42 consecutive days would be very difficult to implement in practice. However, to increase the reliability of the alcohol reports, a push message was sent each morning asking about alcohol consumption on the previous day, thereby keeping the length of time between the actual behavior and recollection to a minimum. Although it has been argued that self-reported alcohol consumption measures can be reliable and valid [47], especially if the recall covers a short period in time, future research should try to replicate our findings using more objective measures of alcohol use.

A second limitation might be that our study was relatively intensive by asking participants for daily participation in the app and questionnaire over a period of 6 weeks. This may have potentially led to a decrease in engagement at the end of this period. This could also be a potential explanation why there were hardly any effects in phase 2. Although we had no visible dropout at this time and people still logged in daily to the app during the last 3 weeks, we cannot be sure they were as engaged with the app as they were during the first 3 weeks. Potentially, participants paid less attention to the posts in the app (including the alcoholposts), thereby decreasing their potential impact. This might also explain the limited effects of the experimental posts because these only occurred during the last 3 weeks. Future studies could take into account measures of engagement to address such explanations.

Third, our study focused on the influence of alcohol posts on Facebook because this was the most popular platform among our target group and alcohol posts were common on Facebook. However, in recent years, other social media platforms (eg, Snapchat and Instagram) have gained popularity, especially among adolescent users [48]. Although we expect the effects of a single alcoholpost described in this paper to be visible in other social media contexts as well, it might be the case that the effects are even more pronounced on Instagram or Snapchat. On Instagram, pictures tend to have higher quality and are made more attractive by adding filters [16], potentially leading to more positive and appealing alcohol pictures and possible stronger effects. On Snapchat, posts can be shared privately or only appear for a short amount of time. This could potentially lead to more extreme posts being shared (eg, drunken pictures), with possibly stronger effects on drinking behavior. Future research is therefore needed to investigate how our findings on the effects of alcoholposts translate to other platforms.

Fourth, another limitation is that the SNS app only included posts from college students. Therefore, it is possible that the posts were more homogenous than they would be on a real Facebook timeline, which may also include posts from, for example, family members. However, we did see that the posts in the tool covered many different topics aside from alcohol. Furthermore, in real life, young people mostly interact with their peers on social media (and generally tend to avoid their parents), and Facebook’s algorithm ensures that they will especially see posts by like-minded individuals [49]. Nevertheless, although we think that the SNS app resembled real life to a sufficient degree, it might be improved by also including posts from diverse individuals leading to a more heterogenous social media environment.

### Conclusion

This study shows a clear and direct effect of exposure to alcoholposts on next-day alcohol consumption. Seeing a natural alcoholpost increases whether young people drink the following day as well as the number of drinks they consume. Although these effects were less visible in the second phase of the study when experimental alcoholposts were also present, these findings suggest that alcoholposts represent an important societal problem that future interventions need to address. Furthermore, the finding that campaigns increased the chance of a drinking day regardless of whether the campaign post was positive (ie, an alcohol commercial) or negative (ie, an antialcohol campaign) about alcohol is relevant for campaign planners and should be further explored in future research.

## Appendix

Multimedia Appendix 1 Translation of posts in Figure 2.

Multimedia Appendix 2 Additional information on Bayesian approach.

Multimedia Appendix 3 Additional tables.

## Abbreviations

alcoholpost: alcohol-related SNS content
CI: credible interval
SNS: social networking site
