# Supplementary material for: Causal Effects of Alcohol-Related Facebook Posts on Drinking Behavior: Longitudinal Experimental Study
Source: J Med Internet Res. 2021 Nov 11;23(11):e28237. doi: 10.2196/28237 (PMC8663476; doi:10.2196/28237)
Supplement: Multimedia Appendix 1 [file jmir_v23i11e28237_app1.docx]

**Multimedia Appendix 1.** Translation of posts in Figure 2.

Positive social posts, from left to right: *“We always have fun, drinking at Paddy’s 😊” (a bar); “Yes, concert-time! Looking forward to it!” ; “Lovely night out with my girlfriends! Cheers!”; link to Desperados ad; link to Bacardi ad; News article entitled “Research shows that couples who drink wine together are happier”.*

Positive non-social posts, from left to right: *“Time for some wine! Nice to relax 😊”; “Nice to go out to dinner!”; “Enjoying relaxation with a book and a drink”; link to Heineken ad; link to captain Morgan ad; News article entitled “Alcohol is good for your endurance”.*

Negative social posts, from left to right: *“Enough alcohol for tonight: it’s not going well ☹”; “Damn, the night ended badly! Had too much to drink…”; “Shit man, feeling ill. That’s what happens with too much booze ☹”; link to anti-alcohol campaign; link to anti-alcohol campaign; News article entitled “Alcohol abuse increases the chance of harm in the following month”.*

Negative non-social posts, from left to right: *“This was a bad idea, I feel really bad ☹”; “Drank too much last night. Regretting that now, I am losing the whole day. Hangover!”; “Ugh, regretting yesterday’s booze, headache!”; link to anti-alcohol campaign; link to anti-alcohol campaign; News article entitled “Binge drinking is bad for the bones of youth”.*
