# Supplementary material for: Causal Effects of Alcohol-Related Facebook Posts on Drinking Behavior: Longitudinal Experimental Study
Source: J Med Internet Res. 2021 Nov 11;23(11):e28237. doi: 10.2196/28237 (PMC8663476; doi:10.2196/28237)
Supplement: Multimedia Appendix 2 [file jmir_v23i11e28237_app2.docx]

Multimedia Appendix 2. Additional information on Bayesian approach.

All multilevel models are estimated using weakly informative priors: a wide normal distribution for intercepts and coefficients of predictors and a decomposition of the covariance matrices. All models are based on an effective number of iterations that is sufficiently large to indicate convergence of the estimation process, namely 4000 draws with as many draws in the warm-up phase. Posterior checks do not indicate estimation problems for the models reported here except that the mean and standard deviation of the number of glasses of alcohol drunk tend to be overestimated by the negative binomial models.
