# Supplementary material for: Causal Effects of Alcohol-Related Facebook Posts on Drinking Behavior: Longitudinal Experimental Study
Source: J Med Internet Res. 2021 Nov 11;23(11):e28237. doi: 10.2196/28237 (PMC8663476; doi:10.2196/28237)
Supplement: Multimedia Appendix 3 [file jmir_v23i11e28237_app3.docx]

**Multimedia Appendix 3.** Additional tables.

Table S1. *Unstandardized regression coefficients with their 95% credible (posterior) intervals for the models predicting alcohol use occurrence and quantity from exposure to alcohol posts published on the preceding day. Bayesian estimation with the R rstanarm package.*

| Dependent variable | Occurrence of Alcohol Use (Y/N) | | |  | Quantity of Alcohol Use (number of glasses) | | |
| --- | --- | --- | --- | --- | --- | --- | --- |
|  |  | 95% Credible Interval | |  |  | 95% Credible Interval | |
| Parameter | b | Lower limit | Upper limit |  | B | Lower limit | Upper limit |
| **Fixed Effects** |  |  |  |  |  |  |  |
| (Intercept) | -1.83 | -2.34 | -1.33 |  | -1.23 | -1.64 | -0.83 |
| Exposure to experimental alcohol posts | 0.04 | -0.03 | 0.11 |  | 0.00 | -0.06 | 0.06 |
| Exposure to natural alcohol posts | 0.27 | 0.18 | 0.35 |  | 0.21 | 0.14 | 0.29 |
| Exposure natural * Phase | -0.35 | -0.48 | -0.22 |  | -0.27 | -0.38 | -0.15 |
| Phase | 0.57 | 0.41 | 0.72 |  | 0.44 | 0.31 | 0.58 |
| Female | -0.43 | -0.74 | -0.11 |  | -0.38 | -0.63 | -0.13 |
| Education (in years) | 0.08 | -0.03 | 0.18 |  | 0.03 | -0.05 | 0.12 |
| Alcohol use frequency | 0.02 | 0.01 | 0.03 |  | 0.01 | 0.01 | 0.02 |
| Alcohol usual quantity | 0.05 | -0.01 | 0.10 |  | 0.12 | 0.08 | 0.16 |
| Monday | -0.64 | -0.93 | -0.38 |  | -0.86 | -1.17 | -0.59 |
| Tuesday | -0.41 | -0.71 | -0.14 |  | -0.61 | -0.91 | -0.33 |
| Wednesday | 0.28 | 0.00 | 0.54 |  | 0.27 | 0.02 | 0.52 |
| Thursday | 0.59 | 0.37 | 0.80 |  | 0.70 | 0.48 | 0.90 |
| Friday | 1.51 | 1.29 | 1.74 |  | 1.41 | 1.22 | 1.60 |
| Saturday | 1.31 | 1.09 | 1.54 |  | 1.33 | 1.15 | 1.51 |
| **Random Effects** (σ) |  |  |  |  |  |  |  |
| Participants Monday variation | 0.73 | 0.29 | 1.34 |  | 1.25 | 0.72 | 2.01 |
| Participants Tuesday variation | 0.74 | 0.34 | 1.37 |  | 1.07 | 0.60 | 1.75 |
| Participants Wednesday variation | 0.58 | 0.14 | 1.18 |  | 0.56 | 0.22 | 1.01 |
| Participants Thursday variation | 0.08 | 0.00 | 0.39 |  | 0.24 | 0.01 | 0.60 |
| Participants Friday variation | 0.22 | 0.00 | 0.59 |  | 0.00 | 0.00 | 0.06 |
| Participants Saturday variation | 0.53 | 0.20 | 0.97 |  | 0.01 | 0.00 | 0.12 |
| Participants within-group intercept variation | 0.59 | 0.45 | 0.78 |  | 0.29 | 0.21 | 0.40 |
| Group intercept variation | 0.15 | 0.03 | 0.34 |  | 0.13 | 0.06 | 0.27 |
| *N* Groups | 49 |  |  |  | 49 |  |  |
| *N* Participants | 281 |  |  |  | 281 |  |  |
| *N* Alcohol reports | 8794 |  |  |  | 8789 |  |  |
| All models estimated with weakly informative priors: a wide normal distribution for intercepts and coefficients of predictors, and a decomposition of the covariance matrices. All models are based on an effective number of iterations that is sufficiently large to indicate convergence of the estimation process, namely 4,000 draws with as many draws in the warm-up phase. Posterior checks do not indicate estimation problems for the models reported here except that the mean and standard deviation of the number of glasses of alcohol drunk tend to be overestimated by the negative binomial models. | | | | | | | |

Table S2. *Unstandardized regression coefficients with their 95% credible (posterior) intervals for the models predicting alcohol use occurrence and quantity from exposure to alcohol posts published on the preceding day, distinguishing between positive and negative experimental (fake) posts. Bayesian estimation with the R rstanarm package.*

| Dependent variable | Occurrence of Alcohol Use (Y/N) | | |  | Quantity of Alcohol Use (number of glasses) | | |
| --- | --- | --- | --- | --- | --- | --- | --- |
|  |  | 95% Credible Interval | |  |  | 95% Credible Interval | |
| Parameter | b | Lower limit | Upper limit |  | b | Lower limit | Upper limit |
| **Fixed Effects** |  |  |  |  |  |  |  |
| (Intercept) | -1.76 | -2.29 | -1.23 |  | -1.19 | -1.60 | -0.77 |
| Exposure to fake alcohol posts (mc) | 0.02 | -0.06 | 0.10 |  | -0.02 | -0.10 | 0.05 |
| Exposure experimental (mc) * Positive | 0.04 | -0.05 | 0.14 |  | 0.05 | -0.04 | 0.13 |
| Positive | -0.09 | -0.43 | 0.24 |  | -0.10 | -0.37 | 0.18 |
| Exposure to natural alcohol posts | 0.27 | 0.18 | 0.35 |  | 0.22 | 0.15 | 0.29 |
| Exposure natural * Phase | -0.35 | -0.47 | -0.23 |  | -0.27 | -0.38 | -0.16 |
| Phase | 0.57 | 0.42 | 0.72 |  | 0.44 | 0.31 | 0.58 |
| Female | -0.41 | -0.73 | -0.11 |  | -0.37 | -0.61 | -0.14 |
| Education (in years) | 0.08 | -0.03 | 0.18 |  | 0.03 | -0.05 | 0.12 |
| Alcohol use frequency | 0.02 | 0.01 | 0.03 |  | 0.01 | 0.01 | 0.02 |
| Alcohol usual quantity | 0.05 | -0.01 | 0.10 |  | 0.12 | 0.08 | 0.16 |
| Monday | -0.64 | -0.93 | -0.38 |  | -0.86 | -1.17 | -0.58 |
| Tuesday | -0.41 | -0.70 | -0.14 |  | -0.61 | -0.89 | -0.33 |
| Wednesday | 0.28 | 0.01 | 0.52 |  | 0.28 | 0.04 | 0.51 |
| Thursday | 0.58 | 0.37 | 0.80 |  | 0.70 | 0.48 | 0.91 |
| Friday | 1.50 | 1.28 | 1.74 |  | 1.41 | 1.22 | 1.60 |
| Saturday | 1.31 | 1.09 | 1.54 |  | 1.33 | 1.16 | 1.51 |
| **Random Effects** |  |  |  |  |  |  |  |
| Participants Monday variation | 0.72 | 0.30 | 1.35 |  | 1.23 | 0.69 | 1.97 |
| Participants Tuesday variation | 0.73 | 0.30 | 1.36 |  | 1.08 | 0.60 | 1.75 |
| Participants Wednesday variation | 0.57 | 0.13 | 1.16 |  | 0.55 | 0.23 | 0.99 |
| Participants Thursday variation | 0.09 | 0.00 | 0.40 |  | 0.25 | 0.00 | 0.59 |
| Participants Friday variation | 0.22 | 0.00 | 0.59 |  | 0.00 | 0.00 | 0.05 |
| Participants Saturday variation | 0.54 | 0.22 | 0.97 |  | 0.01 | 0.00 | 0.11 |
| Participants within-group intercept variation | 0.59 | 0.44 | 0.77 |  | 0.29 | 0.21 | 0.40 |
| Group intercept variation | 0.16 | 0.03 | 0.36 |  | 0.14 | 0.06 | 0.28 |
| *N* Groups | 49 |  |  |  | 49 |  |  |
| *N* Participants | 281 |  |  |  | 281 |  |  |
| *N* Alcohol reports | 8794 |  |  |  | 8789 |  |  |
| All models estimated with weakly informative priors: a wide normal distribution for intercepts and coefficients of predictors, and a decomposition of the covariance matrices. All models are based on an effective number of iterations that is sufficiently large to indicate convergence of the estimation process, namely 4,000 draws with as many draws in the warm-up phase. Posterior checks do not indicate estimation problems for the models reported here except that the mean and standard deviation of the number of glasses of alcohol drunk tend to be overestimated by the negative binomial models.  (mc): this variable is mean-centered. | | | | | | | |

Table S3. *Unstandardized regression coefficients with their 95% credible (posterior) intervals for the models predicting alcohol use occurrence and quantity from exposure to alcohol posts published on the preceding day, distinguishing between social and other experimental (fake) posts. Bayesian estimation with the R rstanarm package.*

| Dependent variable | Occurrence of Alcohol Use (Y/N) | | |  | Quantity of Alcohol Use (number of glasses) | | |
| --- | --- | --- | --- | --- | --- | --- | --- |
|  |  | 95% Credible Interval | |  |  | 95% Credible Interval | |
| Parameter | b | Lower limit | Upper limit |  | B | Lower limit | Upper limit |
| **Fixed Effects** |  |  |  |  |  |  |  |
| (Intercept) | -1.82 | -2.37 | -1.26 |  | -1.21 | -1.65 | -0.78 |
| Exposure to experimental alcoholposts (mc) | 0.02 | -0.06 | 0.11 |  | 0.00 | -0.07 | 0.07 |
| Exposure experimental (mc) * Social | 0.03 | -0.07 | 0.13 |  | 0.00 | -0.09 | 0.09 |
| Social post | 0.04 | -0.29 | 0.37 |  | -0.04 | -0.34 | 0.25 |
| Exposure to natural alcohol posts | 0.27 | 0.18 | 0.36 |  | 0.22 | 0.14 | 0.29 |
| Exposure natural * Phase | -0.35 | -0.47 | -0.23 |  | -0.27 | -0.38 | -0.16 |
| Phase | 0.57 | 0.42 | 0.72 |  | 0.44 | 0.31 | 0.58 |
| Female | -0.42 | -0.74 | -0.12 |  | -0.38 | -0.63 | -0.14 |
| Education (in years) | 0.08 | -0.03 | 0.18 |  | 0.03 | -0.05 | 0.11 |
| Alcohol use frequency | 0.02 | 0.01 | 0.03 |  | 0.01 | 0.01 | 0.02 |
| Alcohol usual quantity | 0.05 | -0.01 | 0.10 |  | 0.12 | 0.08 | 0.16 |
| Monday | -0.64 | -0.92 | -0.40 |  | -0.86 | -1.15 | -0.59 |
| Tuesday | -0.41 | -0.72 | -0.13 |  | -0.60 | -0.93 | -0.31 |
| Wednesday | 0.28 | 0.02 | 0.53 |  | 0.27 | 0.02 | 0.51 |
| Thursday | 0.58 | 0.37 | 0.80 |  | 0.70 | 0.49 | 0.90 |
| Friday | 1.51 | 1.28 | 1.73 |  | 1.41 | 1.22 | 1.60 |
| Saturday | 1.32 | 1.08 | 1.55 |  | 1.34 | 1.15 | 1.52 |
| **Random Effects** |  |  |  |  |  |  |  |
| Participants Monday variation | 0.73 | 0.29 | 1.36 |  | 1.24 | 0.71 | 1.99 |
| Participants Tuesday variation | 0.75 | 0.30 | 1.35 |  | 1.08 | 0.60 | 1.76 |
| Participants Wednesday variation | 0.56 | 0.11 | 1.16 |  | 0.56 | 0.23 | 1.02 |
| Participants Thursday variation | 0.09 | 0.00 | 0.41 |  | 0.25 | 0.01 | 0.59 |
| Participants Friday variation | 0.22 | 0.00 | 0.59 |  | 0.00 | 0.00 | 0.05 |
| Participants Saturday variation | 0.55 | 0.22 | 0.98 |  | 0.01 | 0.00 | 0.12 |
| Participants within-group intercept variation | 0.59 | 0.44 | 0.78 |  | 0.29 | 0.21 | 0.40 |
| Group intercept variation | 0.16 | 0.03 | 0.35 |  | 0.14 | 0.06 | 0.28 |
| *N* Groups | 49 |  |  |  | 49 |  |  |
| *N* Participants | 281 |  |  |  | 281 |  |  |
| *N* Alcohol reports | 8794 |  |  |  | 8789 |  |  |
| All models estimated with weakly informative priors: a wide normal distribution for intercepts and coefficients of predictors, and a decomposition of the covariance matrices. All models are based on an effective number of iterations that is sufficiently large to indicate convergence of the estimation process, namely 4,000 draws with as many draws in the warm-up phase. Posterior checks do not indicate estimation problems for the models reported here except that the mean and standard deviation of the number of glasses of alcohol drunk tend to be overestimated by the negative binomial models.  (mc): this variable is mean-centered. | | | | | | | |

Table S4. *Unstandardized regression coefficients with their 95% credible (posterior) intervals for the models predicting alcohol use occurrence and quantity from exposure to different types of experimental (fake) alcohol posts published on the preceding day. Bayesian estimation with the R rstanarm package.*

| Dependent variable | Occurrence of Alcohol Use (Y/N) | | |  | Quantity of Alcohol Use (number of glasses) | | |
| --- | --- | --- | --- | --- | --- | --- | --- |
|  |  | 95% Credible Interval | |  |  | 95% Credible Interval | |
| Parameter | b | Lower limit | Upper limit |  | b | Lower limit | Upper limit |
| **Fixed Effects** |  |  |  |  |  |  |  |
| (Intercept) | -1.75 | -2.28 | -1.25 |  | -1.23 | -1.66 | -0.80 |
| Exposure to experimental campaign posts | 0.16 | 0.06 | 0.26 |  | 0.03 | -0.05 | 0.12 |
| Exposure to experimental news posts | -0.06 | -0.17 | 0.05 |  | -0.05 | -0.16 | 0.05 |
| Exposure to experimental personal posts | 0.02 | -0.07 | 0.11 |  | 0.00 | -0.08 | 0.08 |
| Exposure to natural alcohol posts | 0.24 | 0.15 | 0.33 |  | 0.21 | 0.14 | 0.29 |
| Exposure natural * Phase | -0.33 | -0.46 | -0.20 |  | -0.26 | -0.37 | -0.15 |
| Phase | 0.53 | 0.38 | 0.69 |  | 0.43 | 0.29 | 0.56 |
| Female | -0.42 | -0.73 | -0.11 |  | -0.38 | -0.62 | -0.13 |
| Education (in years) | 0.08 | -0.03 | 0.18 |  | 0.03 | -0.05 | 0.12 |
| Alcohol use frequency | 0.02 | 0.01 | 0.03 |  | 0.01 | 0.01 | 0.02 |
| Alcohol usual quantity | 0.05 | -0.01 | 0.10 |  | 0.12 | 0.08 | 0.16 |
| Monday | -0.61 | -0.91 | -0.33 |  | -0.84 | -1.13 | -0.55 |
| Tuesday | -0.45 | -0.76 | -0.17 |  | -0.61 | -0.92 | -0.33 |
| Wednesday | 0.23 | -0.03 | 0.50 |  | 0.27 | 0.01 | 0.51 |
| Thursday | 0.51 | 0.27 | 0.74 |  | 0.68 | 0.44 | 0.90 |
| Friday | 1.37 | 1.11 | 1.63 |  | 1.38 | 1.17 | 1.60 |
| Saturday | 1.26 | 1.04 | 1.49 |  | 1.32 | 1.13 | 1.51 |
| **Random Effects** |  |  |  |  |  |  |  |
| Participants Monday variation | 0.71 | 0.28 | 1.37 |  | 1.23 | 0.72 | 1.99 |
| Participants Tuesday variation | 0.71 | 0.29 | 1.34 |  | 1.08 | 0.59 | 1.75 |
| Participants Wednesday variation | 0.56 | 0.12 | 1.16 |  | 0.57 | 0.27 | 1.03 |
| Participants Thursday variation | 0.09 | 0.00 | 0.41 |  | 0.24 | 0.01 | 0.59 |
| Participants Friday variation | 0.25 | 0.01 | 0.62 |  | 0.00 | 0.00 | 0.06 |
| Participants Saturday variation | 0.53 | 0.21 | 0.97 |  | 0.01 | 0.00 | 0.11 |
| Participants within-group intercept variation | 0.59 | 0.44 | 0.79 |  | 0.30 | 0.21 | 0.40 |
| Group intercept variation | 0.16 | 0.03 | 0.36 |  | 0.13 | 0.05 | 0.28 |
| *N* Groups | 49 |  |  |  | 49 |  |  |
| *N* Participants | 281 |  |  |  | 281 |  |  |
| *N* Alcohol reports | 8794 |  |  |  | 8789 |  |  |
| All models estimated with weakly informative priors: a wide normal distribution for intercepts and coefficients of predictors, and a decomposition of the covariance matrices. All models are based on an effective number of iterations that is sufficiently large to indicate convergence of the estimation process, namely 4,000 draws with as many draws in the warm-up phase. Posterior checks do not indicate estimation problems for the models reported here except that the mean and standard deviation of the number of glasses of alcohol drunk tend to be overestimated by the negative binomial models. | | | | | | | |
